# Iodine Intake Estimation from the Consumption of Instant Noodles, Drinking Water and Household Salt in Indonesia

**DOI:** 10.3390/nu10030324

**Published:** 2018-03-08

**Authors:** Aang Sutrisna, Jacky Knowles, Abas Basuni, Ravi Menon, Anung Sugihantono

**Affiliations:** 1 Global Alliance for Improved Nutrition (GAIN), Menara Palma 7th Floor, Jln. HR Rasuna Said Blok X2 kav 6, Jakarta 12950, Indonesia; jacky@jackyknowlesconsultancy.com (J.K.); rmenon@gainhealth.org (R.M.); 2 National Institute for Health Research and Development, Jln. Percetakan Negara No. 29, Jakarta 10560, Indonesia; abas1952@gmail.com; 3 Ministry of Health Indonesia Direktorat Jenderal Kesehatan Masyarakat, Jln. HR. Rasuna Said Blok X-5 Kav. 4-9, Jakarta 12950, Indonesia; sugihantonoa@yahoo.com

**Keywords:** modelling iodine intake, iodine intake, iodised salt, food industry, Indonesia

## Abstract

The objective of this study was to assess the contribution of iodine intake from iodised household salt, iodised salt in instant noodles, and iodine in ground water in five regions of Indonesia. Secondary data analysis was performed using the 2013 Primary Health Research Survey, the 2014 Total Diet Study, and data from food industry research. Iodine intake was estimated among 2719 children, 10–12 years of age (SAC), 13,233 women of reproductive age (WRA), and 578 pregnant women (PW). Combined estimated iodine intake from the three stated sources met 78%, 70%, and 41% of iodine requirements for SAC, WRA and PW, respectively. Household salt iodine contributed about half of the iodine requirements for SAC (49%) and WRA (48%) and a quarter for PW (28%). The following variations were found: for population group, the percentage of estimated dietary iodine requirements met by instant noodle consumption was significantly higher among SAC; for region, estimated iodine intake was significantly higher from ground water for WRA in Java, and from household salt for SAC and WRA in Kalimantan and Java; and for household socio-economic status (SES), iodine intake from household salt was significantly higher in the highest SES households. Enforcement of clear implementing regulations for iodisation of household and food industry salt will promote optimal iodine intake among all population groups with different diets.

## 1. Introduction

Iodine is an element essential for mammalian life; it is absorbed by the thyroid gland where it is converted into thyroid hormones which are required for growth and development throughout life. Iodine is especially important for ensuring optimal foetal brain development during pregnancy [1,2,3]. The World Health Organization (WHO) recommended daily nutrient intake (RNI) for iodine is 90 µg for children 0–5 years of age, 120 µg for school age children (SAC) 6–12 years of age, 150 µg for children and adults 12 years of age and over, and 250 µg for pregnant women (PW) and lactating women [1,4]. Universal Salt Iodisation (USI) has been recommended by WHO and UNICEF as the most cost-effective public health strategy for the prevention of iodine deficiency disorders (IDD). A target of 90% households using adequately iodised salt was set by WHO, UNICEF and the International Council for Control of IDD to indicate achievement of USI. While optimal iodine intake is defined at the population level by a median urinary iodine concentration (MUIC) of 100–299 μg/L, 100–199 μg/L, 150–249 μg/L and ≥100 μg/L for SAC, women of reproductive age (WRA), PW and lactating women, respectively [4].

Iodine deficiency in Indonesia was previously assessed to be a moderate to severe problem, based on the prevalence of goiter [5]. Therefore, national legislation for USI was introduced to combat the problem in 1994 [6]. Indonesian standards define adequately iodised household salt as salt with >30 mg/kg potassium iodate, equivalent to approximately 18 mg/kg iodine [7]. Based on a quantitative iodine analysis in salt samples from the Indonesia Primary Health Research Surveys—Riset Kesehatan Dasar (Riskesdas)—in 2007 and 2013, the percentage of households using salt with adequate iodine content decreased from 62.8% of households in 2007, to 55.1% in 2013 [8,9]. However, the percentage of households using salt with at least some added iodine (>5 mg/kg iodine) was 92% in both years. Despite household coverage with adequately iodised salt being well below the international target of 90%, population level iodine status was found to be within the optimal range in the 2013 Riskesdas. The MUICs for SAC, WRA, PW and lactating women were 215 μg/L, 190 μg/L, 163 μg/L and 164 μg/L, respectively [8]. The previous Riskesdas survey in 2007 also reported adequate iodine status among SAC, with the MUIC found to be 224 μg/L [9], indicating that optimal iodine intake had been maintained among this group.

Although correlation analyses from Riskesdas 2013 indicated some association between iodine intake (assessed as UIC) and the level of iodine in household salt on a national level [10], the district and national median UICs were still higher than might be expected if additional dietary iodine was from the iodisation of household salt only. This statement is based on the low coverage of adequately iodised household salt and known shifts in dietary habits away from the consumption of household salt. Nationally, the Ministry of Trade and National Bureau of Statistics data show a progressive shift in salt supply from cooking and table salt to food industry salt, which has become increasingly apparent since 2011 [11]. This is in line with the country’s rapid transformation to an urban economy [12,13]. Urbanization, increasing incomes, and associated changing lifestyles are known to be driving a demand shift in Indonesia and other countries in Asia towards processed foods [14,15], along with an associated expansion of food processing, procurement and retailing, economic growth and foreign investment, and mass media [16,17].

It was therefore postulated that other sources of dietary iodine, including food sources of iodised salt, were likely contributing to iodine status findings in the 2013 survey. In Indonesia, there is widespread consumption of noodles which are known to be largely manufactured with iodised salt [18]. There are also reports of iodine in ground water (non-packaged drinking water) consumed by household members.

The Indonesian Presidential Decree Number 69/1994 on USI requires the use of iodised salt in all foods for human and animal consumption and in the food and animal feed industries [6]. However, there are currently no implementation regulations for the food industry component of this decree which means that the food industry’s use of iodised salt is not monitored and is likely to be inconsistent. An investigation of food industry practices was conducted by PT Clarity Research Indonesia in 2013 with the support of the Ministry of Health, the National Agency of Drug and Food Control (BPOM) and the Global Alliance for Improved Nutrition (GAIN) [18]. The main findings from industrially processed food producers who participated in the study were that iodised salt was being used in the production of over 90% of the market share of chilli sauce and bread (about 10% of the total bread market) and in the production of approximately two thirds of the market share of instant noodles. Based on consumption data, instant noodles were the largest contributor to dietary salt intake of the products investigated, which also included food seasoning products, biscuits and soy sauce.

This paper reports on data from the 2013 Riskesdas and the 2014 Total Diet Study [19] (Survei Konsumi Makanan Individu, SKMI), which was conducted in a sub-sample of approximately 69% of Riskesdas households. This paper assesses and describes the relative contributions of household salt, instant noodles and non-packaged drinking water as dietary sources of iodine among school-age children, 10–12 years of age, WRA, and PW in Indonesia. It aims to provide this information to the national programme for the elimination of iodine deficiency.

## 2. Materials and Methods

Estimation of iodine intake from different sources was conducted through a secondary analysis of data from the cross-sectional Riskesdas and SKMI, combined with additional information on instant noodles from supermarket product surveys, the PT Clarity Research report [18], and different investment reports, to estimate market share [20,21]. Estimation of iodine intake from instant noodle consumption was conducted by multiplied estimated iodine content from the PT Clarity Research report and product survey with the frequency of consumption from Riskesdas. Estimation of iodine intake from household salt and non-packaged drinking water was conducted by multiplied estimated daily consumption from SKMI with iodine content from Riskesdas.

The sampled population for both Riskesdas and SKMI was households in 33 provinces and 497 districts/cities in Indonesia. The SKMI 2014 included 51,127 households, selected using random systematic selection from the 294,959 households in the total Riskesdas 2013 sample. An independently selected subset of 11,430 households from Riskesdas 2013 was targeted for the collection of household salt and quantitative analysis of iodine content. A further subset of 3028 households was targeted for non-packaged drinking water. The iodine content of salt and water was assessed using titration [22,23]. Estimates of iodine intake from the consumption of instant noodles, non-packaged drinking water, and household salt were calculated for 2719 SAC, 10–12 years of age, 13,233 WRA and 578 PW. These estimates were calculated for the respective population groups resident in the Riskesdas households selected for the collection and analysis of household salt.

The socio-economic status (SES) of each individual in the combined dataset was categorised into three groups based on the calculated wealth quintile index from Riskesdas 2013. The wealth quintile indices 1 and 2 were categorised as the lowest economic status (SES-1) for this study, index 3 was categorised as an intermediate economic status (SES-2) and indices 4 and 5 were categorised as the highest economic status (SES-3).

The typical frequency of instant noodle consumption (number of packets) was available for Riskesdas 2013 respondents who were 10 years of age and above. Iodine intake from instant noodles was estimated using the following steps:

1. Estimation of salt content per packet of instant noodles:
  - Data on typical sodium content and instant noodle packet weight were collected for multiple product types, differing in packet weight and sodium content per unit weight from the two producers: Indofood Sukes Makmur and Wings Group. These producers supply almost 90% of the instant noodle market in Indonesia [20,24] and the products included were from their four best-selling brands [21]. These were three Indofood brands—Indomie (seven product types), Supermi (two types) and Sarimi (two types)—and one Wings group brand: Mi Sedap (four product types).
  - The sodium content per packet was converted to the equivalent average salt content for each of the four brands using a 2.5 conversion factor (sodium contributes approximately 40% to the molecular weight of salt). An assumption was made that salt is the source of almost all sodium in instant noodle packets. Indonesian noodles typically contain monosodium glutamate and sodium compounds as preservatives. It was not possible to determine the exact amount; however, it is relatively small and sodium from salt is the major contributor to total sodium content. The salt content is from both the dried noodle and in the flavouring sachet.
2. Estimation of iodine content per packet of instant noodle. The iodine content per 1 gram of salt in instant noodles was estimated based on available information on whether the producer used iodised salt in the production of noodles and/or the spice mix. Where iodised salt was used, a salt iodine level equivalent to the minimum national salt iodisation standard (18 mg/kg or 18 µg/g) [7] was assumed. Information from the salt supplier’s Certificates of Analysis for 10 instant noodle product lines indicated that salt contained at least 18 mg/kg iodine, and in 40% of samples, the level was greater than 23 mg/kg iodine [18].
3. The average milligrams sodium/packet, grams salt/packet and assumed micrograms iodine/packet were calculated after weighting the results for each brand by the market share for that brand, scaled up to assume that these brands covered 100% of the market.
4. Categorisation of the weekly amount (number of packets) of instant noodles typically consumed was conducted by taking the reported frequency of instant noodles consumption and converting it into a category of likely weekly consumption. The reported frequency of consumption options from the questionnaire were >1 time/day, 1 time/day, 3–6 times/week, 1–2 times/week, and <3 times/month; these were assigned to the categories 10, 7, 4.5, 1.5, and 0.5 packages per week, respectively. The conversion from >1 packet/day to 10 packets per week was based on the reported average of 1.48 packets/day consumed by SKMI respondents who consumed instant noodles every day (1.48 × 7 = 10.4) [19].

Based on the average weekly consumption estimates, the associated potential iodine intakes (from point 2) were calculated and used to estimate the percent of the recommended nutrient intake (RNI) for iodine that could be met for each population group.

The Wings group did not participate in the PT Clarity study; therefore, this paper used the conservative assumption that Wings group noodles (Mi Sedap) were not produced with iodised salt. The discussion section includes comments on current knowledge about this (gained after the analyses in this manuscript were completed) and the implications for the overall contribution of instant noodles to dietary iodine intake.

Twenty-four hour dietary recall data from SKMI were used to calculate the median non-packaged water (mL/day) and the median salt (g/day) consumption for SAC, WRA and PW for each of the following five regions: Sumatra, Java, Kalimantan, Sulawesi and Bali-Papua.

The analysed iodine content of non-packaged drinking water was available for a sub-set of Riskesdas households; however, the available data did not allow for links to be made with households in the SKMI dataset. Therefore, the median iodine content of non-packaged drinking water by region was applied to all households without the result in the respective region. The regional median household non-packaged water iodine content was multiplied by the mean daily quantity of non-packaged water consumed for each population group to obtain an estimate of typical daily iodine intake from non-packaged drinking water by population group within each region.

SES data were only available within the available Riskesdas dataset; therefore, the median household iodine content of non-packaged drinking water by SES group (from Riskesdas) was multiplied by the median daily consumption of non-packaged water for each population group in the region where the household was located (from SKMI).

Iodine intake from household salt for each population group by region and SES group was estimated using a similar method as for water. The analysed iodine content of household salt was available for a large sub-set of Riskesdas households; however, as above, it was not possible to link these households to the households in the SKMI dataset. Therefore, the regional median iodine content of salt (from Riskesdas) was applied to the household without the result and multiplied by the median daily quantity of household salt consumed (from SKMI) for each population group in the same region to obtain an estimate of typical daily iodine intake from household salt by population group and region. In addition, the median iodine content of household salt by SES group (from Riskesdas) was multiplied by the median typical daily consumption of household salt (from SKMI) for the region where the household was located.

Statistical analyses were conducted using Stata Statistical Software: Release 12 (StataCorp. 2011, College Station, TX, USA). Final analyses were adjusted with consideration of the sample design and associated data weighting [8]. Normality was tested using the Shapiro–Wilks test. Descriptive statistics were used to present the outcome variables, using medians and inter-quartile ranges. The significance of differences between groups was assessed based on non-overlapping 95% confidence intervals around the mean. For regional analyses of all data for PW and data for non-packaged water iodine for SAC, the sample size for several regions was less than 50. In these cases, results are only shown for the national level estimates.

## 3. Results

### 3.1. Estimated Iodine Intake from the Consumption of Instant Noodles

Table 1 shows the outcome of the retail market-based survey for the salt and iodine contents of the leading brands of instant noodles, accounting for approximately 87% of the market. The sodium content of the instant noodles from supermarket product surveys ranged between 1095 and 1308 mg per packet. An overall average of 1256 mg sodium was determined after adjusting for the respective market share of each brand and scaling up to 100% of the market. The average salt content was estimated to be 3.14 g/packet, with an associated average iodine content of 46.5 µg per packet, based on data available at the time of the analyses.

Table 2 models the estimated daily iodine intake from instant noodles and the related percentage of RNI for iodine for each population group, according to the consumption frequency category. For respondents who reported consuming instant noodles more than once a day, this intake potentially contributed to over half (55.3%) of the RNI for iodine for SAC, almost 44.3% of the RNI for non-pregnant WRA and just over a quarter (26.6%) of the RNI for PW.

The median reported instant noodle intake among SAC was 3–6 packets per week (data not shown), which is estimated to meet around one quarter of the daily iodine needs for this population group. The median reported intake among both WRA and PW was 1–2 times per week, estimated to meet around 7% (WRA) and 4% (PW) of the daily iodine needs for these groups (Table 2).

The estimated mean and median iodine intakes from instant noodles by region and SES group for each population group are shown in Table 3 and Table 4, respectively. Differences in intake are driven by differences in the frequency of consumption, since the same average iodine content per packet is used throughout. The mean estimated daily intake of iodine from the consumption of instant noodles was 23 µg/day for SAC, 18.2 µg/day for WRA and 16.2 µg/day for PW, which is equivalent to 19.2%, 12.1% and 6.5% of the population group-specific daily RNIs for iodine, respectively (Table 3). When the population group-specific percent RNI was based on the median intake, the percent was similar to that for the frequency of consumption (25%, 7% and 4%, respectively).

There was no significant difference in the mean estimated iodine intake from instant noodles by region among SAC (Table 3). However, SAC from households in the SES-2 category had higher intakes (mean 24.9 µg/day; 95% CI 23.6, 26.2) than those from households in the SES-1 category (mean 21.8 µg/day; 95% CI 20.7, 22.8) (Table 4). Among WRA, the mean estimated iodine intake from instant noodles was lower in the Sumatra region (mean 16.4 µg/day; 95% CI 15.8, 16.9) than in all other regions. It was also lower in the Java region (mean 18.2 µg/day; 95% CI 17.8, 18.5) than in Kalimantan, Sulawesi and Bali-Papua (Table 3). No significant differences in iodine intake from instant noodles by household SES category were observed for WRA or for PW (Table 4).

### 3.2. Estimated Iodine Intake from the Consumption of Non-Packaged Drinking Water

Table 5 shows the average non-packaged water iodine content, average consumption, and related estimated iodine intake from non-packaged water for each of the five regions for WRA, and as national estimates for SAC and PW. For WRA, the mean analysed iodine content of non-packaged drinking water was significantly lower in Sumatra (19.6 µg/L; 95% CI 17.2, 22.0) and Kalimantan (15.6 µg/L; 95% CI 12.7, 18.5) than in Java (24.7 µg/L; 95% CI 22.3, 27.1). The mean analysed content of non-packaged water by region was slightly different for SAC, since it was based on information from a different (lower) number of households. However, it followed a similar pattern of relative iodine content by region (data not shown).

For SAC, daily consumption of non-packaged drinking water ranged from 692 mL/day in Bali-Papua (significantly lower than in the other four regions) to 874 mL/day in Java (data not shown), with an overall mean consumption of 840 mL/day. For WRA, consumption ranged from 931 mL/day in Bali-Papua (significantly lower than in the other four regions) to 1104 mL/day in Java, with an overall mean of 1072 mL/day. For PW, the overall mean consumption of non-packaged drinking water was 1147 mL/day.

Table 5 indicates that the estimated mean iodine intake from non-packaged drinking water among SAC was 11.7 µg iodine/day, equivalent to just under 10% of the RNI for iodine for this population group. The estimated mean iodine intake from non-packaged drinking water for both WRA and PW was 16.7 µg/day, equivalent to 11.1% of the RNI for iodine for WRA and 6.7% of the RNI for PW.

The estimated daily iodine intake from non-packaged drinking water varied by region for WRA. The estimated iodine intake from water in Bali-Papua was significantly lower (mean 9.7 µg/day; 95% CI 9.0, 10.5) than in other regions, while iodine intake from non-packaged drinking water was significantly higher in Java (mean 16.5 µg/day; 95% CI 16.0, 17.0) than in other regions.

Table 6 shows no significant differences in the mean iodine intake from non-packaged drinking water by household SES category among SAC or PW. However, iodine intake from non-packaged drinking water increased significantly with household SES category among WRA, from 13.7 µg iodine/day (95% CI 13.4, 14.0) for SES-1 households to 17.1 µg iodine/day (95% CI 16.5, 17.7) for SES-3 households.

### 3.3. Estimated Iodine Intake from Salt Consumption

The findings for the iodine content of household salt (Table 7) for SAC and WRA showed a fairly consistent pattern by region. A significantly higher mean household salt iodine content was found in the Kalimantan region for SAC (30.2 mg/kg; 95% CI 26.5, 34.0) and WRA (27.3 mg/kg; 95% CI 25.7, 28.9) than for other regions. The mean household salt iodine content was lowest in the Sumatra region for SAC (16.6 mg/kg; 95% CI 14.9, 18.2) and WRA (16.5 mg/kg; 95% CI 16.0, 17.1). Among SAC, the difference was significant when compared with the levels in Kalimantan and Java. Among WRA, the level was significantly lower than in all other regions.

The reported household salt consumption data resulted in average salt intakes which were fairly comparable between regions. Higher intakes were reported among WRA and PW (mean salt intake 3.7 and 3.6 g/day, respectively, for the national sample) than among SAC (mean salt intake 3.0 g/day for the national sample).

Estimated iodine intake and the percentage of the RNI for iodine from household salt showed the same regional trends as for salt iodine content for SAC and WRA. The intake of iodine from household salt was significantly higher in the Kalimantan region (93.1 mg/kg; 95% CI 82.6, 103.6 for SAC and 100.2 mg/kg; 95% CI 95.0, 105.4 for WRA) than in all other regions (except for Sulawesi for WRA). Household salt iodine in this region potentially accounted for 77.6% of the iodine RNI for SAC and 66.8% of the RNI for WRA. The lowest contributions to dietary intake of iodine through household salt were found in Sumatra, where it accounted for approximately 37% of the RNI for both SAC and WRA.

Overall, household salt iodine was estimated to have provided approximately half of the RNI for iodine in the national sample of SAC (48.8% of the RNI) and WRA (47.6%) and just over a quarter of the RNI for the national sample of PW (28.0% of the RNI).

Table 8 shows that estimated salt consumption was similar between household SES categories for each population group. The iodine content of household salt was, however, found to be 2.4 to 5.8 mg/kg lower for SES-1 households than for SES-3 households—a significant difference for each population group. This resulted in significantly lower iodine intakes from household salt for all three population groups in the lowest SES category households when compared with the same group in the highest SES category households. The percentages of the iodine RNI consumed in household salt for households in the SES-1 category were 44.5% for SES-1 and 52.4% for SES-3 for SAC; compared with households in the SES-3 category, which showed percentages of 44.1% and 49.8% for WRA, and 23.1% and 29.8% for PW.

### 3.4. Estimated Iodine Intake from the Three Sources Combined

**Figure 1:**
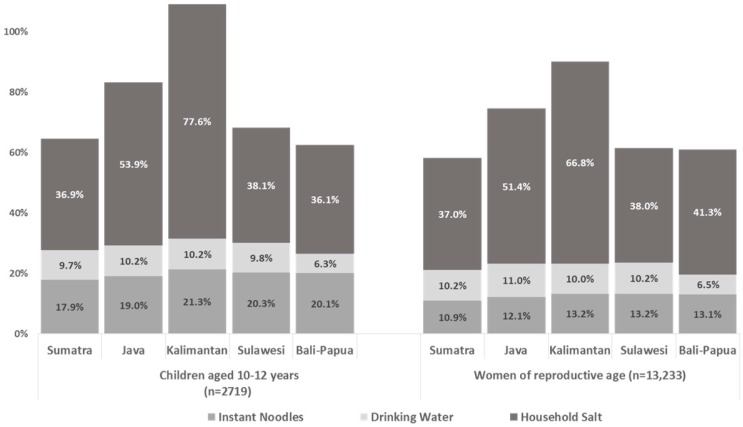
Estimates for the percent contributions to the iodine recommended nutrient intake (RNI) from iodised salt in instant noodles, household salt iodine and from trace iodine in non-packaged drinking water, according to respondent group and region.

Estimates of combined iodine intake from each of the three products above—instant noodles, non-packaged drinking water, and household salt—were calculated for each sub-group to estimate the potential combined contribution to the iodine RNI. Figure 1 illustrate the total combined contribution with an indication of individual source contributions for each population group by region and by household SES category.

For all groups, iodine from household salt can be seen to be the most important of the three sources contributing to dietary iodine intake; this source also shows the most variation between sub-groups.

The total estimated iodine intake from these three sources accounted for 63% (Bali-Papua region) to 109% (Kalimantan region) of the iodine RNI for SAC, and 58% (Sumatra region) to 90% (Kalimantan region), of the iodine RNI for WRA (Figure 1). The second highest regional iodine intakes from these three sources (after Kalimantan) were found in the Java region, accounting for approximately 83% of the iodine RNI for SAC and 75% of the RNI for WRA. The estimated combined contributions to the iodine RNI were approximately the same—58–68% of the RNI—for the remaining three regions for both SAC and WRA.

**Figure 2:**
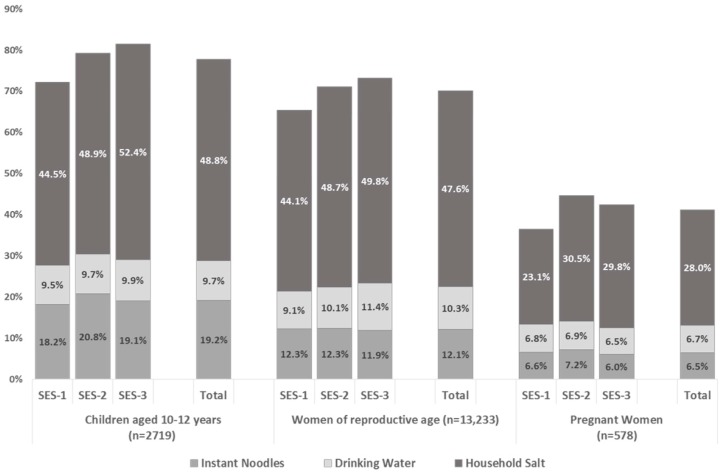
Estimates for the percent contribution to the iodine recommended nutrient intake (RNI) from iodised salt in instant noodles, household salt iodine and from trace iodine in non-packaged drinking water, according to respondent group and household socio-economic status (SES). SES-1 indicates the lowest status, SES-3 indicates the highest status. The estimates are based on iodine RNIs of 120 µg, 150 µg and 250 µg for SAC, WRA, and PW, respectively [22].

Figure 2 indicates that, among SAC and WRA, consumption of these three products has an increasing contribution to iodine intake as household SES increases. The potential contribution to the iodine RNI changed from 72.2% among SAC from households in the SES-1 category to 81.5% among SAC from households in the SES-3 category. A similar increase, from 65.4 to 73.1% of the iodine RNI, was seen among WRA from households in the SES-1 and SES-3 categories. A similar but less pronounced increase in iodine intake from these three sources was seen among PW, increasing from 36.5 (SES-1) to 42–45% (SES-2 and 3) of the estimated contribution to the iodine RNI.

Figure 2 also shows the estimated total iodine intake and contribution to the iodine RNI for instant noodles, non-packaged drinking water and household salt on a national level. These were estimated to be 93.2 µg/day and 77.7% of the iodine RNI for SAC; 105.2 µg/day and 70.1% of the RNI for WRA; and 102.9 µg/day and 41.2% of the RNI for PW.

## 4. Discussion

This study shows that, on a national level in Indonesia, iodine intake from iodine added to household salt, together with iodine added to salt used in the production of instant noodles and the naturally occurring iodine from non-packaged drinking water, meets around three quarters of the iodine requirements for SAC and WRA, and around two fifths of requirements among PW. PW had the lower percentage of iodine RNI met by all sources, due, at least partially, to the higher recommended requirement for this group. On a sub-national level, these three dietary sources met over half the iodine RNI for SAC and WRA in all five regions.

Of the three sources, iodine from household salt consumption provided the greatest relative contribution to iodine intake among all respondent groups. These data suggest that without iodisation of salt in the household and food industry (in this case instant noodles), dietary intake of iodine may be below the RNI across all population groups. Additional studies of other (non-salt, non-water) sources of dietary iodine, combined with an assessment of the relationship between urinary sodium and urinary iodine are needed to fully determine this.

The assumptions used for the estimation of the iodine content of instant noodles in this paper were conservative in terms of salt iodine level and industry practices regarding iodised salt. Evidence generated since the time of the survey and data analysis indicate that the Wings group now use iodised salt in the production of Mi Sedap instant noodles [25]. If all other assumptions remain the same, the consideration that Wings noodles use iodised salt would increase the average iodine content per packet of instant noodles from 46.5 µg to 56.5 µg. This increases the relative contribution to dietary iodine from this source by about 20% of its current amount.

The estimated average consumption of salt from various food sources reported from the 2014 SKMI is 6.8 g/day for SAC, WRA and PW. This is higher than the Indonesian Ministry of Health recommended intake of 3.7 g salt/day (based on a recommended intake of sodium of not more than 1500 mg per day) [26] and the WHO recommended intake of 5 g/day. The estimated contributions of household salt to this total salt consumption are 44%, 54% and 52% for SAC, WRA and PW, respectively [19]. Current regulatory guidelines for salt iodisation in Indonesia are only enforced for household salt. If all salt for human consumption was iodised to 18 mg/kg, then consuming 6.8 g of salt a day from household and food industry sources of salt could provide 122 µg dietary iodine. This would meet approximately 100%, 80% and 50% of the recommended iodine requirements for SAC, WRA and PW, respectively. The additional estimated 12–13 µg iodine from non-packaged drinking water would maintain the iodine intakes within an optimal level for SAC and reach over 90% and 60% of dietary needs for WRA and PW, respectively.

Differences in dietary behaviour between the three population groups meant that the intake of iodine from instant noodles was almost double that estimated from non-packaged drinking water among SAC. Among WRA and PW, the relative contributions to dietary iodine from instant noodles and non-packaged drinking water were similar.

In general, the degree to which food industry (iodised) salt contributes to total dietary salt and related iodine intake would be expected to vary within Indonesia. These variations occur according to the national food industry structure and practices of using iodised salt, food distribution and population access to markets, and regional differences in dietary practices. Ensuring regulation for both the use of, and expected acceptable range for, iodised salt in the food industry will help standardise iodine intake regardless of dietary sources of salt.

Current national standards for salt iodisation in Indonesia do not include an upper level of iodisation. This should be reconsidered based on these data, which suggest the average iodine content for household salt in Kalimantan, for example, took total iodine intake to levels above requirements for SAC in this region (a region which accounts for only 6% of the total population). In the meantime, regulatory monitoring of the household salt supply should be strengthened, focused initially on the supply to the Kalimantan and Sumatra regions and to areas of lower SES, where results suggest that the salt supply may have been inadequately iodised.

Enforced iodisation of all sources of edible salt, combined with monitoring iodine status among different population groups will inform and facilitate future adjustments in salt iodine standards. This has particular relevance to salt reduction initiatives which can and should be implemented in a complementary manner with USI [27].

The quantity of non-packaged drinking water consumed and its iodine content were both highly variable. The range of iodine levels in non-packaged drinking water was found to be 0 to 439 µg/L, with a median of 17 µg/L in urban areas and 13 µg/L in rural areas, nationally [8]. It would not be feasible or recommended to adjust salt iodisation standards at the sub-national level to account for these variations. It is, however, helpful to have an idea of the degree of variation, which may, in some cases, help to explain notable discrepancies between iodine status and access to iodised household and processed food salt.

The limitations of this study were mainly due to constraints regarding the available data from the SKMI dataset. The fact that it was not possible to link households between surveys meant it was necessary to apply regional estimates for non-packaged drinking water and household salt to households in a region. In addition, the exact proportion of sodium from salt in the instant noodles could not be verified; however, it was assumed that the amount from preservatives and sodium-containing flavour enhancers was a small proportion of the total. The design, methodological concept and results are, however, valuable as this is the first investigation of this kind in Indonesia.

## 5. Conclusions

The results presented in this paper show that using household coverage of adequately iodised salt as an indicator of progress towards USI and optimal iodine nutrition in Indonesia currently captures the major dietary source of iodine. However, these data and the background referenced literature make it clear that household salt iodine content and coverage cannot be used as the only indicators of total iodine intake from iodised salt sources, or as a reliable proxy for expected iodine status. Household salt does not account for all dietary sources of (potentially iodised) salt, especially among SAC and among the population in lower SES households. These data add to a body of evidence supporting the development of additional programme guidance to better advocate for iodisation of all edible salt, including in processed foods and condiments, with associated regulations and monitoring [28,29,30].

It is clear that iodisation of all edible salt will provide iodine through a variety of dietary sources, ensuring more equitable protection from inadequate iodine intake. Iodisation in the food industry as well as in household salt is particularly important for those living in areas without access to adequately iodised household salt and for population groups consuming an increasing proportion of dietary salt from processed foods rather than from household salt [12,31].

This study also provides answers to a long-standing question about the variation in ground water iodine levels in Indonesia and the influence this may have on iodine status. The estimated contribution of non-packaged drinking water iodine to the regional average for iodine intake, while widely variable, did not reach higher than 11% of the RNI for iodine for any sub-group. This would therefore not be expected to have a major impact on population iodine status when compared with that from USI.

## Figures and Tables

**Table 1:** Estimated salt and iodine contents of the most popular brands of instant noodles.

Instant Noodle Brand	2013 Estimated Market Share *	Average Sodium (mg)/Packet Instant Noodles	Estimated Salt (g)/Packet Instant Noodles	Iodine µg/g Salt in Instant Noodles ^†^	Iodine µg/Packet Instant Noodles
Indomie	69.0%	1251	3.13	18.0	56.3
Mi Sedap	14.8%	1308	3.27	0.0	0.0
Supermi	1.8%	1095	2.74	18.0	49.3
Sarimi	1.0%	1125	2.81	18.0	50.6
Total market share	86.6%				
Average for the four brands ^‡^		1256	3.14	14.9	46.5

* Based on JP Morgan data for 2013 market share and Top Brand breakdown of the relative share for each of the three Indofood brands [18,21]; ^†^ Estimates are based on the reported use of iodised salt (no available information for Mi Sedap, Wings Group at the time of the analysis) and an assumed level of salt iodine that meets the national minimum standard (18 mg/kg iodine), verified by Certificates of Analysis at the time of the research [18]; ^‡^ Averages were adjusted to represent 100% of the market.

**Table 2:** Estimated iodine intake from instant noodles according to consumption frequency (reported in Riskesdas 2013).

Consumption Frequency	Number of Packets/Week	Estimated Iodine Intake (µg) from Instant Noodle Consumption *		Percent Daily RNI for Iodine for Given Frequency of Consumption ^†^		
		Per Week	Per Day	SAC	WRA	PW
>1 time/day	10	464.6	66.4	55.3%	44.3%	26.6%
1 time/day	7	325.2	46.5	38.7%	31.0%	18.6%
3–6/week	4.5	209.1	29.9	24.9%	19.9%	11.9%
1–2/week	1.5	69.7	10.0	8.3%	6.6%	4.0%
<3×/month	0.5	23.2	3.3	2.8%	2.2%	1.3%
Never	0	0	0	0.0%	0.0%	0.0%

RNI, recommended nutrient intake; SAC, school aged children, 10–12 years of age; WRA, non-pregnant women of reproductive age; PW, pregnant women; * Based on an average of 46.5 µg iodine per packet of instant noodles; ^†^ Based on RNIs of 120 µg, 150 µg and 250 µg iodine for SAC, WRA and PW, respectively [22].

**Table 3:** Mean and median estimated intakes of iodine (µg/day) from the consumption of instant noodles for each respondent group by geographic region.

	*N*	Estimated Daily Iodine Intake (µg) from Instant Noodles *				Percent Iodine RNI ^‡^
		Mean ^†^	95% CI	Median	IQR	
SAC						
Sumatra	506	21.5	20.1–22.8	10.1	10.1–30.3	17.9%
Java	1581	22.9	22.0–23.6	30.3	10.1–30.3	19.0%
Kalimantan	136	25.5	22.2–28.7	30.3	10.1–47.1	21.3%
Sulawesi	230	24.3	22.1–26.5	30.3	10.1–30.3	20.3%
Bali-Papua	266	24.1	21.9–26.3	20.2	10.1–30.3	20.1%
Total	2719	23.0	22.3–23.6	30.3	10.1–30.3	19.2%
WRA						
Sumatra	2501	16.4 ^a^	15.8–16.9	10.1	10.1–30.3	10.9%
Java	7956	18.2 ^b^	17.8–18.5	10.1	10.1–30.3	12.1%
Kalimantan	694	19.8 ^c^	18.5–21.0	10.1	10.1–30.3	13.2%
Sulawesi	899	19.8 ^c^	18.7–21.0	10.1	10.1–30.3	13.2%
Bali-Papua	1183	19.6 ^c^	18.6–20.6	10.1	10.1–30.3	13.1%
Total	13,233	18.2	17.9–18.4	10.1	10.1–30.3	12.1%
PW						
Total	578	16.2	15.0–17.3	10.1	10.1–30.3	6.5%

95% CI, 95% confidence interval; IQR, interquartile range; RNI, daily recommended nutrient intake; SAC, school aged children 10–12 years of age; WRA, non-pregnant women of reproductive age; PW, pregnant women; * Based on an estimated average of 46.5 µg iodine per packet of instant noodles; ^†^ Significant differences between the means (non-overlapping 95% CI) within a population group are indicated by superscript letters; ^‡^ Based on calculated mean iodine intakes and RNIs of 120 µg, 150 µg and 250 µg iodine for SAC, WRA, and PW, respectively [22].

**Table 4:** Mean and median estimated intakes of iodine (µg/day) from the consumption of instant noodles according to respondent group and household SES category.

	*N*	Estimated Daily Iodine Intake (µg) from Instant Noodles *				Percent Iodine RNI ^‡^
		Mean ^†^	95% CI	Median	IQR	
SAC						
SES-1	962	21.8 ^a^	20.7–22.8	10.1	10.1–30.3	18.2%
SES-2	620	24.9 ^b^	23.6–26.2	30.3	10.1–30.3	20.8%
SES-3	1137	22.9 ^a,b^	22.0–23.8	30.3	10.1–30.3	19.1%
WRA						
SES-1	4368	18.4	17.9–18.8	10.1	10.1–30.3	12.3%
SES-2	2975	18.5	17.9–19.0	10.1	10.1–30.3	12.3%
SES-3	5890	17.9	17.5–18.3	10.1	10.1–30.3	11.9%
PW						
SES-1	172	16.5	14.2–18.8	10.1	10.1–30.3	6.6%
SES-2	135	18.0	15.3–20.7	10.1	10.1–30.3	7.2%
SES-3	271	15.1	13.5–16.7	10.1	10.1–30.3	6.0%

SES, socio-economic status; 95% CI, 95% confidence interval; IQR, interquartile range; RNI, daily recommended nutrient intake; SAC, school aged children 10–12 years of age; WRA, non-pregnant women of reproductive age; PW, pregnant women; * Based on an estimated average of 46.5 µg iodine per packet of instant noodles; **^†^** Significant differences between the means (non-overlapping 95% CI) within a population group are indicated by superscript letters; **^‡^** Based on calculated mean iodine intakes and RNIs of 120 µg, 150 µg and 250 µg iodine for SAC, WRA, and PW, respectively [22].

**Table 5:** Mean and median iodine contents of non-packaged drinking water (µg/L) and estimated daily iodine intakes and percentages of RNI from non-packaged drinking water by region.

	*N*	Analysed Iodine Content (µg/L) of Non-Packaged Drinking Water				*N*	Average Water Consumption (mL/day)	Estimated Daily Iodine Intake (µg) from Non-Packaged Drinking Water				Percent Iodine RNI ^†^
		Mean *	95% CI	Median	IQR			Mean *	95% CI	Median	IQR	
SAC												
Total	484	19.5	17.6–21.4	15.0	0–30	2719	840	11.7	11.3–12.0	10.6	10–12.6	9.7%
WRA												
Sumatra	365	19.6 ^a^	17.2–22.0	16.0	2–27	2501	1051	15.3 ^a^	14.9–15.8	13.5	9.5–16.1	10.2%
Java	1258	24.7 ^b^	22.3–27.1	15.0	2–30	7956	1104	16.5 ^b^	16.0–17.0	12.6	12.6–14.9	11.0%
Kalimantan	105	15.6 ^a^	12.7–18.5	14.0	0–27	694	1033	15.1 ^a^	14.5–15.6	17.1	7.9–18.5	10.0%
Sulawesi	129	21.2 ^a,b^	17.4–25.0	16.0	0–35	899	1062	15.3 ^a^	14.7–16.0	13.9	11.8–18.2	10.2%
Bali-Papua	206	19.6 ^a,b^	16.2–23.0	12.0	0–32	1183	931	9.7 ^c^	9.0–10.5	6.6	0–16.4	6.5%
Total	2063	22.6	21.0–24.2	15.0	0–30	13,233	1072	16.7	15.2–15.8	13.2	12.6–16.3	11.1%
PW												
Total	213	17.3	14.5–20.1	12.0	0–26	578	1147	16.7	15.4–18.0	13.2	10.8–18.3	6.7%

95% CI, 95% confidence interval; IQR, interquartile range; RNI, daily recommended nutrient intake; SAC, school aged children, 10–12 years of age; WRA, non-pregnant women of reproductive age; PW, pregnant women; * Significant differences between the means (non-overlapping 95% CI) within a population group are indicated by superscript letters; **^†^** Based on calculated mean iodine intakes and RNIs of 120 µg, 150 µg and 250 µg iodine for SAC, WRA, and PW, respectively [22].

**Table 6:** Mean and median iodine contents of non-packaged drinking water (µg/L) and estimated daily iodine intakes (µg) and percentages of RNI from non-packaged drinking water by household SES category.

	*N*	Analysed Iodine Content (µg/L) of Non-Packaged Drinking Water				*N*	Average Water Consumption (mL/day)	Estimated Daily Iodine Intake (µg) from Non-Packaged Drinking Water				Percent Iodine RNI ^†^
		Mean *	95% CI	Median	IQR			Mean *	95% CI	Median	IQR	
SAC												
SES-1	159	23.4	19.7–21.2	19	5–32	962	823	11.4	10.8–12.0	10.6	10–11.8	9.5%
SES-2	117	17.3	13.9–20.7	13	0–28	620	842	11.6	11.0–12.2	10.6	10–12.6	9.7%
SES-3	208	17.7	15.0–20.5	12	0–27.5	1137	853	11.9	11.4–12.4	10.6	9.8–12.9	9.9%
WRA												
SES-1	602	18.9 ^a^	17.1–20.7	14	0–29	4368	1052	13.7 ^a^	13.4–14.0	12.6	12.6–15.9	9.1%
SES-2	468	21.1 ^a,b^	17.6–24.6	14	0–28	2975	1069	15.1 ^b^	14.5–15.7	12.6	12.6–16.1	10.1%
SES-3	993	25.6 ^a^	23.0–28.3	16	1–31	5890	1089	17.1 ^c^	16.5–17.7	13.9	12.6–16.9	11.4%
PW												
SES-1	60	19.6	14.3–24.9	18	0–27	172	1114	17.0	14.6–19.3	13.2	11.8–19.4	6.8%
SES-2	49	20.2	13.0–27.4	10	0–38	135	1122	17.3	14.1–20.5	13.2	10.8–17.5	6.9%
SES-3	104	14.7	11.1–18.2	11	0–22.5	271	1181	16.3	14.5–18.0	13.2	10.8–18.3	6.5%

SES, socio-economic status; 95% CI, 95% confidence interval; IQR, interquartile range; RNI, daily recommended nutrient intake; SAC, school aged children 10–12 years of age; WRA, non-pregnant women of reproductive age; PW, pregnant women; * Significant differences between the means (non-overlapping 95% CI) within a population group are indicated by superscript letters; **^†^** Based on calculated mean iodine intakes and RNIs of 120 µg, 150 µg and 250 µg iodine for SAC, WRA, and PW, respectively [22].

**Table 7:** Mean and median iodine contents of household salt (mg/kg) and estimated daily iodine intakes (µg) and percentages of RNI from household salt by region.

	*N*	Analysed Iodine Content (mg/kg) of Household Salt				*N*	Average Household Salt Consumption (g/day)	Estimated Daily Iodine Intake (µg) from Household Salt				Percent Iodine RNI ^†^
		Mean *	95% CI	Median	IQR			Mean *	95% CI	Median	IQR	
SAC												
Sumatra	413	16.6 ^a^	14.9–18.2	13.0	7.4–21.2	506	2.8	44.3 ^a^	40.7–47.8	35.8	21.5–52.2	36.9%
Java	1341	20.6 ^b^	19.8–21.4	16.9	10.6–26.5	1581	3.2	64.7 ^b^	62.5–66.9	55.8	35.4–81.3	53.9%
Kalimantan	115	30.2 ^c^	26.5–34.0	24.3	15.9–39.1	136	3.1	93.1 ^c^	82.6–103.6	80.4	54.4–118.5	77.6%
Sulawesi	185	18.9 ^a,b^	17.0–20.9	15.9	9.5–24.8	230	2.7	45.8 ^a^	42.1–49.5	36.1	29.5–58.4	38.1%
Bali-Papua	235	18.6 ^a,b^	16.5–20.7	13.8	5.3–26.5	266	2.6	43.3 ^a^	39.0–47.6	31.8	17.7–54.7	36.1%
Total	2289	20.0	19.4–20.7	15.9	9.5–26.5	2719	3.0	58.6	56.9–60.3	49.3	30.5 – 74	48.8%
WRA												
Sumatra	2067	16.5 ^a^	16.0–17.1	13.8	8.5–21.2	2501	3.5	55.5 ^a^	53.9–57.1	47.0	29.4–67.4	37.0%
Java	6773	20.6 ^b^	20.3–21.0	16.9	9.5–27.5	7956	3.8	77.0 ^b^	75.8–78.2	69.0	40.8–98.7	51.4%
Kalimantan	595	27.3 ^c^	25.7–28.9	22.2	14.8–33.9	694	3.7	100.2 ^c^	95.0–105.4	83.6	57.4–117.6	66.8%
Sulawesi	696	19.0 ^b^	18.0–20.0	15.9	9.2–26.2	899	3.3	57.0 ^ac^	54.6–59.4	45.8	36.5–72.1	38.0%
Bali-Papua	1044	20.5 ^b^	19.4–21.6	15.9	6.3–28.6	1183	3.4	62.0 ^c^	59.0–65.0	44.8	27–81.3	41.3%
Total	11,175	20.1	19.8–20.4	15.9	9.2–26.5	13,233	3.7	71.5	70.6–72.4	59.2	37.5–90.1	47.6%
PW												
Total	484	20.4	19.0–21.8	15.9	9.2–28.6	578	3.6	70.0	65.7–74.3	59.0	31.9–92	28.0%

95% CI, 95% confidence interval; IQR, interquartile range; RNI, daily recommended nutrient intake; SAC, School aged children 10–12 years of age; WRA, non-pregnant women of reproductive age; PW, pregnant women; * Significant differences between the means (non-overlapping 95% CI) within a population group are indicated by superscript letters; **^†^** Based on calculated mean iodine intakes and RNIs of 120 µg, 150 µg and 250 µg iodine for SAC, WRA, and PW, respectively [22].

**Table 8:** Mean and median iodine contents of household salt (mg/kg) and estimated daily iodine intakes (µg) and percentages of RNI from household salt by household SES category.

	*N*	Analysed Iodine Content (mg/kg) of Household Salt				*N*	Average Household Salt Consumption (g/day)	Estimated Daily Iodine Intake (µg) from Household Salt				Percent Iodine RNI ^†^
		Mean *	95% CI	Median	IQR			Mean *	95% CI	Median	IQR	
SAC												
SES-1	796	18.4 ^a^	17.3–19.5	14.1	7.4–24.3	962	3.0	53.5 ^a^	50.8–56.1	45.9	26.4–67.6	44.5%
SES-2	520	19.7 ^a,b^	18.4–20.9	15.9	9.5–26.5	620	3.1	58.7 ^a,b^	55.4–61.9	50.8	31–75.3	48.9%
SES-3	973	21.5 ^b^	20.4–22.6	18.0	10.6–28.6	1137	3.1	62.9 ^b^	60.1–65.8	50.9	33–80.6	52.4%
WRA												
SES-1	3683	18.7 ^a^	18.2–19.2	14.8	7.4–25.3	4368	3.7	66.1 ^a^	64.6–67.6	56.6	32.8–81.7	44.1%
SES-2	2482	20.3 ^b^	19.7–20.9	16.7	9.2–26.5	2975	3.7	73.0 ^b^	71.1–75.0	60.8	38.4–91.9	48.7%
SES-3	5010	21.1 ^b^	20.7–21.5	18.0	10.6–27.5	5890	3.7	74.7 ^b^	73.3–76.0	60.8	39.7–96.6	49.8%
PW												
SES-1	142	16.1 ^a^	14.1–18.0	13.9	6.3–21.2	172	3.6	57.8 ^a^	51.1–64.5	48.7	24–78.9	23.1%
SES-2	107	22.9 ^b^	19.4–26.4	20.1	10.6–29.6	135	3.6	76.2 ^b^	66.5–86.0	67.5	35.7–93.3	30.5%
SES-3	235	21.9 ^b^	19.8–23.9	16.9	9.5–31.7	271	3.5	74.6 ^b^	68.2–80.9	59.7	35.4–99.4	29.8%

SES, socio-economic status; 95% CI, 95% confidence interval; IQR, interquartile range; RNI, daily recommended nutrient intake; SAC, school aged children 10–12 years of age; WRA, non-pregnant women of reproductive age; PW, pregnant women; * Significant differences between the means (non-overlapping 95% CI) within a population group are indicated by superscript letters; **^†^** Based on calculated mean iodine intakes and RNIs of 120 µg, 150 µg and 250 µg iodine for SAC, WRA, and PW, respectively [22].
